# Quality Control MIC Ranges Used for Telavancin with Application of a Revised CLSI Reference Broth Microdilution Method

**DOI:** 10.1128/JCM.01210-14

**Published:** 2014-09

**Authors:** James E. Ross, Rodrigo E. Mendes, Ronald N. Jones

**Affiliations:** JMI Laboratories, North Liberty, Iowa, USA

## Abstract

The telavancin broth microdilution susceptibility testing method was revised, which provides MIC results lower than those obtained by the previous method. This study was performed to reestablish the quality control ranges for telavancin when tested against the strains (updated MIC range) Staphylococcus aureus ATCC 29213 (0.03 to 0.12 μg/ml), Enterococcus faecalis ATCC 29212 (0.03 to 0.12 μg/ml), and Streptococcus pneumoniae ATCC 49619 (0.004 to 0.015 μg/ml).

## TEXT

Telavancin is a lipoglycopeptide antibiotic approved in the United States and Canada for the treatment of patients with complicated skin and skin structure infections due to susceptible Gram-positive pathogens and in the United States and Europe for the treatment of hospital-acquired bacterial pneumonia, including ventilator-associated bacterial pneumonia (HABP/VABP) due to susceptible isolates of Staphylococcus aureus (methicillin-resistant Staphylococcus aureus [MRSA] strains only in Europe), when alternative medicines are unsuitable (1). Telavancin is active against nearly all clinically important Gram-positive bacteria: staphylococci (including methicillin-resistant and vancomycin-intermediate strains), streptococci (including multidrug-resistant pneumococci), enterococci (vancomycin-susceptible isolates only), Gram-positive anaerobes such as members of the genus Clostridium (including Clostridium difficile), and other less commonly encountered Gram-positive pathogens (2, 3).

Previous CLSI supplemental documents updated annually (M100-S15 through M100-S23 [4]) recommended the use of dimethyl sulfoxide (DMSO) and water as solvent and diluent, respectively, for preparations of stock solutions and drug dilutions for manufacturing 96-well frozen-form panels for telavancin susceptibility testing. This method was used to establish the previous telavancin MIC quality control (QC) ranges (4). However, the broth microdilution (BMD) method for telavancin has been revised, and now consists of using DMSO as the solvent and the diluent, following the CLSI guidelines for stock solution and dilution preparations of water-insoluble agents (see Table 8B in reference 5). Moreover, similar to dalbavancin and oritavancin, this modified method for telavancin includes the addition of polysorbate-80 (P-80) (0.002%) to the Mueller-Hinton test medium to minimize drug-binding loss to plastic surfaces (6–8).

These changes were shown to improve the solubility of the drug during panel preparation (DMSO) and drug availability in the 96-well plastic plates (P-80), resulting in a more accurate and reproducible *in vitro* assessment of telavancin MIC determinations (Theravance, Inc., data on file [study no. THV-08-001, study no. THV-07-001, and report 09-6424-MCB-01]). In addition, studies conducted during the development of the revised method demonstrated that the MICs for telavancin when tested against staphylococci and enterococci were 4- to 8-fold lower than those obtained by the previous CLSI-recommended method (Theravance, Inc., data on file [report 08-THE-03]). The differences in the telavancin MIC values obtained by both methods were less pronounced when tested against streptococci. Therefore, this study was conducted to reestablish the MIC QC ranges for telavancin when utilizing the revised BMD method.

The study presented here was performed according to the guidelines found in the CLSI M23-A3 document (9), which specifies the use of at least seven laboratories and three different manufacturers of media. This study utilized eight laboratories and four different media lots. Vancomycin was included as a control agent. BMD frozen-form panels were manufactured according to the modified method by Thermo Fisher Scientific (formerly TREK Diagnostics Systems/Sensititre, Cleveland, OH, USA). Results are presented as QC MIC ranges (μg/ml) for three strains (S. aureus ATCC 29213, Enterococcus faecalis ATCC 29212, and Streptococcus pneumoniae ATCC 49619). The telavancin MIC testing range for S. aureus and E. faecalis was 0.008 to 8 μg/ml, and that for S. pneumoniae was 0.001 to 1 μg/ml.

The eight laboratories were experienced clinical and/or research microbiology facilities and followed the CLSI procedures for BMD methods (10). The participating sites (and their principal investigator) were Massachusetts General Hospital, Boston, MA (M. J. Ferraro), Wheaton Franciscan Laboratory, Wauwatosa, WI (E. Munson), JMI Laboratories, North Liberty, IA (R. N. Jones), Thermo Fisher Scientific, Cleveland, OH (C. Knapp), University of Alberta, Edmonton, Alberta, Canada (R. Rennie), University of Washington, Seattle, WA (S. Swanzy), Duke University Medical Center, Durham, NC (S. Mirrett), and Robert Wood Johnson Medical School, New Brunswick, NJ (M. Weinstein). Inoculum colony counts (CFU/ml) were performed on drug-free agar media and resulted in average counts of 2.8 × 10^5^ CFU/ml (S. aureus ATCC 29213), 2.8 × 10^5^ CFU/ml (E. faecalis ATCC 29212), and 2.5 × 10^5^ CFU/ml (S. pneumoniae ATCC 49619). All results were considered acceptable.

The telavancin MIC ranges obtained against ATCC QC strains by the revised and previous BMD methods are summarized in Table 1. Telavancin displayed MIC results between 0.03 and 0.12 μg/ml, with an overall modal MIC value of 0.06 μg/ml (71.3% of MIC values) when tested against S. aureus ATCC 29213 (Tables 1 and 2). These telavancin modal MIC and MIC ranges were 4-fold lower and one doubling dilution narrower than those documented when using the previous method (modal MIC value of 0.25 μg/ml [62.9% of MIC values] and MIC range of 0.12 to 1 μg/ml) (4; S. Brown, minutes of the NCCLS Antimicrobial Susceptibility Subcommittee meeting, 11 January 2004, Tempe, AZ). Additional data analysis was performed using the Range Finder program (12), which confirmed the modal MIC and proposed a QC range for S. aureus of 0.03 to 0.12 μg/ml. Telavancin MIC results obtained against the E. faecalis ATCC 29212 strain were also within a 3-log_2_ dilution range (0.03 to 0.12 μg/ml) with a modal MIC value of 0.06 μg/ml; results confirmed by the Range Finder program (12).

**TABLE 1:** Quality control MIC ranges for telavancin when tested against ATCC strains using the previous and revised broth microdilution methods

QC organism	MIC (μg/ml)	No. (%) of replicates inhibited at telavancin MIC in^*a*^:	
		Revised method^*b*^	Previous method^*c*^
S. aureus ATCC 29213	1		**17 (7.1)**
	0.5		**71 (29.6)**
	0.25		**151 (62.9)**
	0.12	**3 (0.9)**	**1 (0.4)**
	0.06	**228 (71.3)**	
	0.03	**89 (27.8)**	
E. faecalis ATCC 29212	1		1 (0.4)
	0.5		**34 (14.2)**
	0.25		**203 (84.6)**
	0.12	**51 (15.9)**	**2 (0.8)**
	0.06	**265 (82.8)**	
	0.03	**4 (1.3)**	
S. pneumoniae ATCC 49619	0.03		**1 (0.4)**
	0.015	**45 (14.1)**	**22 (9.2)**
	0.008	**268 (83.8)**	**116 (48.3)**
	0.004	**7 (2.2)**	**101 (42.1)**
	0.002		

a Results in bold type represent the MIC QC ranges for the revised susceptibility testing method approved by the CLSI (document M100-S24 [5]) and the previous method (document M100-S23 [4] and the earlier versions [documents M100-S15 through M100-S22]).

b MIC QC ranges for the revised method proposed and approved by the CLSI (document M100-S24[5]). All values were within the proposed ranges.

c MIC QC ranges proposed for the previous method were 0.12 to 1 μg/ml (S. aureus ATCC 29213) (all MICs within range), 0.12 to 0.5 μg/ml (E. faecalis ATCC 29212) (99.6% of MICs within range), and 0.002 to 0.015 μg/ml (S. pneumoniae ATCC 49619) (99.6% of MICs within range) (S. Brown, minutes of the NCCLS Antimicrobial Susceptibility Subcommittee meeting, 11 January 2004, Tempe, AZ).

**TABLE 2:** Medium lot and inter- and intra-laboratory comparisons of telavancin MIC results obtained when tested against the listed ATCC QC strains.

ATCC strain and MIC	No. of occurrences for medium lot:				No. of occurrences for laboratory:								Total no.
	A	B	C	D	A	B	C	D	E	F	G	H	
S. aureus 29213													
0.03 μg/ml	11	30	11	37	12	27		7	8	8	16	11	89
0.06 μg/ml	68	50	67	43	28	12	40	33	31	31	24	29	228
0.12 μg/ml	1		2			1			1	1			3
Total	80	80	80	80	40	40	40	40	40	40	40	40	320
Mode	0.06	0.06	0.06	0.06	0.06	0.03	0.06	0.06	0.06	0.06	0.06	0.06	0.06
Geomean	0.06	0.05	0.06	0.04	0.05	0.04	0.06	0.05	0.05	0.05	0.05	0.05	0.05
Log_2_ dilution range	3	2	3	2	2	3	1	2	3	3	2	2	3
E. faecalis 29212													
0.03 μg/ml				4		2		1				1	4
0.06 μg/ml	73	71	49	72	38	37	37	31	35	23	28	36	265
0.12 μg/ml	7	9	31	4	2	1	3	8	5	17	12	3	51
Total	80	80	80	80	40	40	40	40	40	40	40	40	320
Mode	0.06	0.06	0.06	0.06	0.06	0.06	0.06	0.06	0.06	0.06	0.06	0.06	0.06
Geomean	0.06	0.06	0.08	0.06	0.06	0.06	0.06	0.07	0.07	0.08	0.07	0.06	0.07
Log_2_ dilution range	2	2	2	3	2	3	2	3	2	2	2	3	3
S. pneumoniae 49619													
0.004 μg/ml	2	2	3			1	6						7
0.008 μg/ml	67	72	70	59	23	33	29	34	33	40	40	36	268
0.015 μg/ml	11	6	7	21	17	6	5	6	7			4	45
Total	80	80	80	80	40	40	40	40	40	40	40	40	320
Mode	0.008	0.008	0.008	0.008	0.008	0.008	0.008	0.008	0.008	0.008	0.008	0.008	0.008
Geomean	0.009	0.008	0.008	0.010	0.011	0.009	0.008	0.009	0.009	0.008	0.008	0.009	0.009
Log_2_ dilution range	3	3	3	2	2	3	3	2	2	1	1	2	3

S. pneumoniae ATCC 49619 had telavancin MIC results within 0.004 to 0.015 μg/ml, with an overall modal MIC value of 0.008 μg/ml. These telavancin MIC results obtained against S. pneumoniae ATCC 49619 with the revised method suggested a QC range and modal MIC value similar to those obtained with the previous testing methodology (0.004 to 0.03 μg/ml and 0.008 μg/ml, respectively) (Table 1). However, a greater reproducibility (83.8% versus 48.3% of MIC values at 0.008 μg/ml obtained by the revised and previous methods, respectively) and narrower range was obtained using the revised methodology. The Range Finder calculations confirmed the absence of outlier results, and the tool also suggested an MIC QC range of 0.004 to 0.015 μg/ml. All broth medium lots and laboratories shared the same modal MIC value, regardless of the ATCC strain tested (except for laboratory B versus S. aureus ATCC 29213, where the mode was 0.03 μg/ml [Table 2]). All vancomycin results were within published QC ranges providing a valid internal control (data not shown).

Overall, the updated MIC QC ranges described in this study are narrower (and more reproducible for S. aureus and S. pneumoniae ATCC strains) than those published for telavancin in the M100-S23 and earlier documents (4). Similar observations were reported for dalbavancin and oritavancin, when comparable method modifications were applied to the MIC testing for those agents (6–8). These updated telavancin QC ranges were approved by CLSI and published in the CLSI M100-S24 (2014) document (5). In addition, these data were reviewed and approved as part of a labeling supplement for the product Vibativ (telavancin) by the U.S. Food and Drug Administration. The revised testing method and the QC MIC ranges presented here, as well as updated interpretive breakpoints, have been included in the updated labeling (1). The reestablished QC ranges and associated breakpoints should now be applied for interpretations of telavancin MIC results obtained against QC and clinical isolates when using this revised CLSI BMD method (1, 5).
